# Use of the Subcutaneous Triptorelin Stimulation Test for Diagnosis of Central Precocious Puberty

**DOI:** 10.3390/children10111830

**Published:** 2023-11-20

**Authors:** Jungmin Ahn, Youngin Lee, Seongmin Gim, Hwalrim Jeong

**Affiliations:** 1 Department of Pediatrics, School of Medicine, Jeju National University, Jeju 63243, Republic of Korea; 2 Department of Pediatrics, School of Medicine, Soonchunhyang University Cheonan Hospital, Cheonan 31151, Republic of Korea

**Keywords:** precocious puberty, gonadorelin, triptorelin, GnRH stimulation test

## Abstract

Background: The gold standard gonadotropin-releasing hormone (GnRH) stimulation test uses the response to intravenously injected gonadorelin to diagnose central precocious puberty (CPP). However, gonadorelin is not always readily available. Objective: This study investigated the diagnostic efficacy of the subcutaneous triptorelin test and the optimal blood sampling time for diagnosis of CPP. Methods: This study retrospectively examined the medical records of 220 girls who had undergone either the triptorelin or gonadorelin test and compared their clinical characteristics. We retrospectively compared clinical parameters between girls diagnosed with CPP (*n* = 111) and idiopathic premature thelarche (IPT) (*n* = 109) using three different diagnostic methods: the gonadorelin, triptorelin 120 min, and triptorelin 180 min tests. The diagnostic ability of the stimulated luteinizing hormone (LH) concentration in the triptorelin test for CPP was evaluated using receiver operating characteristic (ROC) analysis. Results: The CPP group exhibited higher basal and peak gonadotropin levels, more advanced bone age, and a lower body mass index standard deviation score than the IPT group. In the gonadorelin test group, all girls with CPP exhibited a peak LH response 30–60 min after intravenous gonadorelin injection. In the triptorelin test group, most girls with CPP exhibited a peak LH response 60–180 min after subcutaneous triptorelin injection (*n* = 68). On the ROC curve, a peak LH concentration of ≥ 4.52 IU/L at 120 min had the highest CPP diagnostic accuracy, with sensitivity and specificity of 100% and 95.83%, respectively.

## 1. Introduction

Precocious puberty is defined as the onset of secondary sexual characteristics before the age of 8 years in girls and 9 years in boys [1]. Puberty is initiated by pulsatile secretion of gonadotropin-releasing hormone (GnRH) and activation of the hypothalamo-pituitary–gonadal (HPG) axis. Central precocious puberty (CPP) is caused by early reactivation of pulsatile GnRH secretion, and treatment with GnRH analogs should be considered. Idiopathic premature thelarche (IPT) is defined as isolated breast development without activation of the HPG axis and bone age (BA) consistent with the chronological age. IPT is a benign and self-limiting condition that does not require therapeutic intervention [2]. Some girls with IPT exhibit significantly advanced BA, which is known to be associated with obesity [3]. This is known as the thelarche variant, a non-progressive form of precocious puberty, but the extent of HPG axis activation and changes therein remains unclear [4]. In this study, we use the term IPT.

Globally, the prevalence of CPP is rising. In the United States and Europe, the reported prevalence of CPP is 1 per 5000–10,000 population [5,6]. In a Korean epidemiologic study, the incidence and prevalence of CPP were higher than those in the United States and Europe. A study that analyzed data from the Korea National Health and Nutrition Examination Survey (KNHANES) 2004–2010 reported a prevalence of 57.6 per 100,000 population and an incidence of 15.9 per 100,000 population; however, an epidemiologic study using data from the KNHANES 2008–2014 reported a steep annual increase, with a prevalence of 193.2 per 100,000 population and an incidence of 122.8 per 100,000 population [7,8]. Furthermore, COVID-19 is thought to have contributed to the increased development of precocious puberty [9,10].

GnRH stimulation is used to confirm activation of the HPG axis and to differentiate between CPP and IPT. Before the COVID-19 pandemic, gonadorelin (Relefact^®^, Sanofi-Aventis, France) was commonly used in the GnRH stimulation test; during the pandemic, however, it became short in supply, and an alternative was needed. Several previous studies have suggested that triptorelin acetate (Decapeptyl 0.1 mg^®^, Ferring, Switerland) could be a reliable alternative to the gold standard GnRH test because its diagnostic accuracy is comparable to that of gonadorelin [11,12,13]. Triptorelin acetate has a longer half-life and stronger affinity for the GnRH receptor than gonadorelin. In addition, because triptorelin is administered subcutaneously and gonadorelin is administered intravenously, the time required for gonadotropin to reach its peak concentration may vary depending on the type of stimulant used. The blood sampling intervals also differ between studies, which has complicated comparisons of their accuracy.

In Korea, the supply of gonadorelin ceased around 2021; therefore, the triptorelin test has since been used as an alternative method to the GnRH test. The triptorelin test measures the gonadotropin level at 0, 30, 60, 90, and 120 min after subcutaneous administration of triptorelin acetate, and CPP is diagnosed when the peak luteinizing hormone (LH) level is ≥5 IU/L. Notably, the gonadorelin test evaluates the LH response after intravenous gonadorelin administration, whereas the triptorelin test assesses the LH response following subcutaneous triptorelin injection. This raises concerns about potential delays in diagnosing precocious puberty due to the delayed increase in LH response; moreover, doubts have been raised as to whether the triptorelin test is as reliable as the gonadorelin test for identifying HPG activation; the appropriate test time also remains unclear. Therefore, we compared the diagnostic utility of the triptorelin test and the gonadorelin test and assessed the optimal blood sampling time for the diagnosis of CPP.

## 2. Materials and Methods

### 2.1. Subjects

This retrospective medical chart review was conducted on 111 girls with idiopathic CPP and 109 girls with IPT who had visited the Pediatric Endocrinology Clinic at Jeju National University Hospital and Soonchunhyang University Cheonan Hospital from 1 March 2020 to 28 February 2023. Age-matched girls with CPP and IPT were included. All girls had exhibited breast enlargement before the age of 8 years. All BAs were at least 1 year ahead of those of the chronological ages. All subjects had undergone either a gonadorelin or triptorelin stimulation test during the day to determine whether the HPG axis was activated. Girls with suspected precocious puberty had undergone the triptorelin test (an alternative diagnostic method) after gonadorelin became unavailable. Girls suspected of precocious puberty at Jeju National University Hospital had undergone the 180 min triptorelin test, and those at Soonchunhyang University Cheonan Hospital had undergone the 120 min test.

Seventy-four girls underwent the conventional GnRH stimulation test using 100 µg gonadorelin. The serum concentrations of LH and follicle-stimulating hormone (FSH) were measured at baseline and at 30, 45, 60, and 90 min after intravenous injection of gonadorelin. In total, 146 girls had undergone the triptorelin test. Of these, 74 girls had undergone the triptorelin test, and the LH and FSH levels were measured at baseline and at 30, 60, 120, and 180 min after subcutaneous injection of triptorelin 0.1 mg (triptorelin 180 min test). Seventy-two girls underwent the triptorelin test, and the LH and FSH levels were measured at baseline and at 30, 60, 90, and 120 min after subcutaneous injection of triptorelin 0.1 mg (triptorelin 120 min test). A peak LH level of ≥5.0 IU/L was considered to indicate HPG axis activation, and CPP was diagnosed. A peak LH level of <5.0 IU/L was classified as IPT. The subjects’ clinical data were collected by retrospective chart review and compared. The study was conducted according to the principles described in the Declaration of Helsinki.

### 2.2. Measurements

The pubertal status (Tanner stage of breast development) of all subjects was assessed and documented by two pediatric endocrinologists. The subjects were categorized based on their pubertal stage (Tanner stage II–V). Height was measured to the nearest 0.1 cm using a Harpenden stadiometer (Holtain, Crosswell, UK). Weight was measured to the nearest 0.1 kg with an electronic scale (CAS Corporation, Seoul, Republic of Korea). BA was assessed by an X-ray of the left hand according to the Greulich–Pyle method [14]. Body mass index (BMI) was calculated as body mass (kg) divided by height squared (m^2^). The standard deviation scores (SDSs) for height, weight, and BMI were calculated using age- and sex-specific least mean square parameters based on 2017 growth reference values for Korean children and adolescents developed by the Korean Pediatric Society and the Korea Centers for Disease Control and Prevention [15].

The LH level was measured using a chemiluminescent microparticle immunoassay (Abbott Diagnostics, Abbott Park, IL, USA). Girls who were small for gestational age or had a chronic illness, chromosomal abnormalities, brain tumors, hypothyroidism, or other endocrinopathies were excluded.

### 2.3. Statistical Analyses

Continuous variables are expressed as mean and standard deviation. Categorical variables are presented as counts. The subjects’ endocrine parameters were compared using Student’s *t*-test and analysis of variance. Correlations between endocrine parameters were evaluated using Pearson’s correlation analysis. The area under the curve for the triptorelin-stimulated LH concentration in the diagnosis of CPP in girls was assessed by receiver operating characteristic (ROC) analysis. A *p* value of <0.05 was used as the cutoff for statistical significance. All data were analyzed using SPSS version 20.0 (IBM Corp., Armonk, NY, USA) and R version 3.5.4 (The R Foundation for Statistical Computing, Austria).

## 3. Results

In total, 220 girls were included in the study. Of these, 146 underwent the triptorelin test, of whom 74 underwent the triptorelin 180 min test, and the other 72 the triptorelin 120 min test. The remaining 74 girls underwent the gonadorelin test. The mean age of all subjects was 8.08 ± 0.68 years (range, 5.16–8.91 years).

Chronological age and height SDS were not significantly different between the CPP and IPT test groups (Table 1). Girls with CPP had significantly higher basal and peak LH and FSH levels than girls with IPT. The peak LH/FSH values of girls with CPP were significantly higher than those of girls with IPT, but the basal LH/FSH levels did not differ significantly. The BA in girls with CPP was significantly higher than that in girls with IPT. Girls with IPT had higher BMI SDSs than girls with CPP.

Pearson’s correlation analysis revealed positive correlations of the Tanner breast stage and BA stage with the peak LH level (*r* = 0.300, *p* < 0.001 and *r* = 0.232, *p* < 0.001, respectively). In addition, the Tanner stage exhibited positive correlations with height SDS and BMI SDS (*r* = 0.307, *p* < 0.001 and *r* = 0.260, *p* < 0.001, respectively).

We analyzed the clinical characteristics of CPP and IPT according to the type of stimulant (gonadorelin vs. triptorelin) (Table 2). Girls with CPP had significantly higher basal LH and peak LH levels than girls with IPT in both the gonadorelin and triptorelin test groups. In both the triptorelin and gonadorelin test groups, girls with CPP exhibited significantly higher basal FSH and peak FSH levels than girls with IPT. The peak LH/FSH values of girls with CPP were significantly higher than those of girls with IPT, but the basal LH/FSH values did not differ significantly. In the overall triptorelin test group, girls with CPP had significantly more advanced BA and a higher mean Tanner breast stage than girls with IPT. However, in the gonadorelin test group, there were no significant differences between girls with CPP and IPT in terms of either BA or the mean Tanner breast stage.

**Figure 1:**
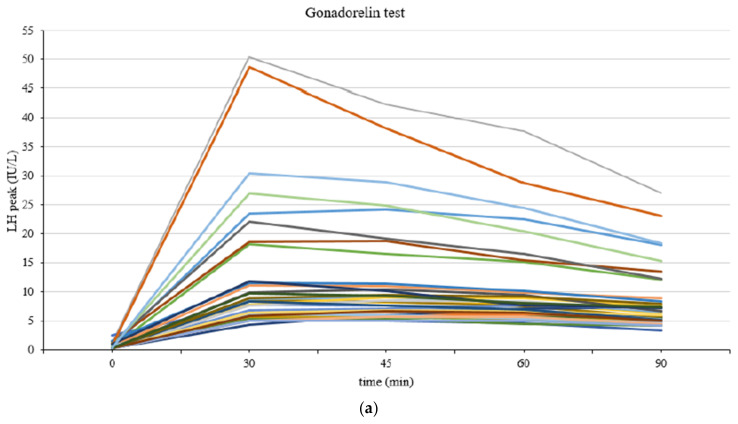

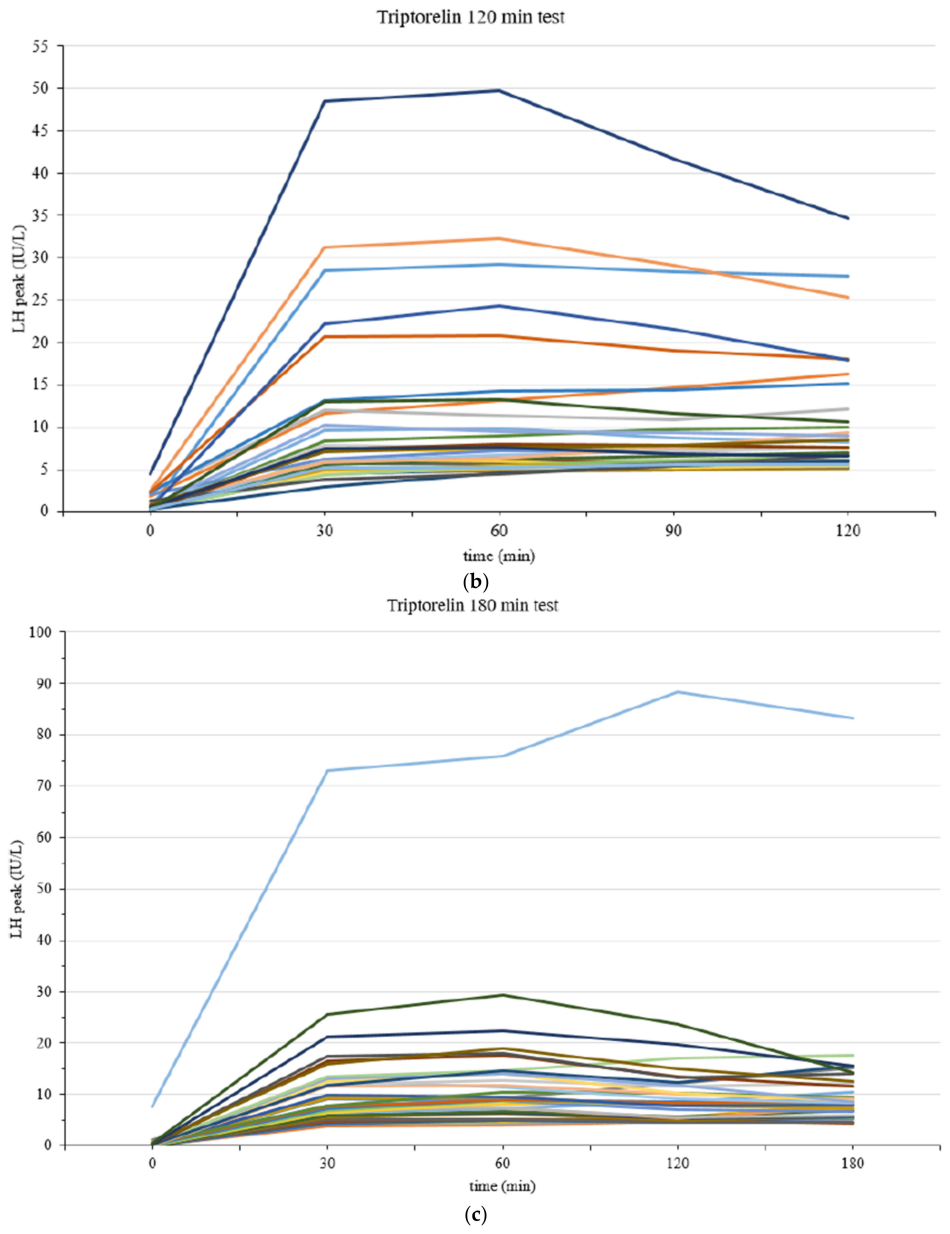

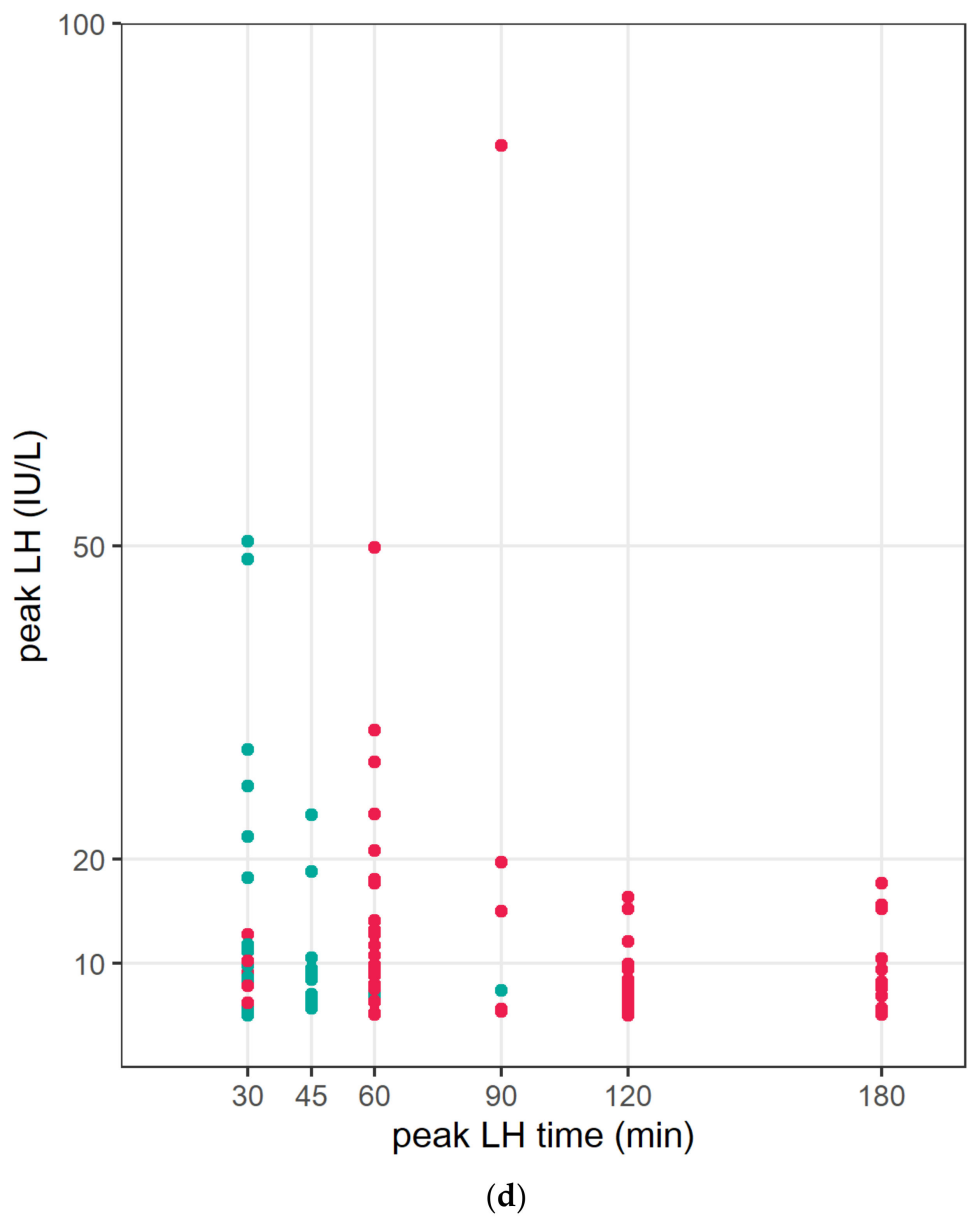
Luteinizing hormone (LH) response in gonadotropin-releasing hormone (GnRH), triptorelin 120 min and triptorelin 180 min tests. (**a**) LH response in the GnRH test. (**b**) LH response in the triptorelin 120 min test. (**c**) LH response in the triptorelin 180 min test. (**d**) Peak LH time according to the type of stimulant given to the CPP group. Green dots represent the time at which the peak LH (luteinizing hormone) is observed in the gonadorelin test, while red dots represent the time at which the peak LH is observed in the triptorelin test.

In the CPP group, the time to reach the peak LH level was longer after triptorelin injection than after gonadorelin injection (Figure 1). In girls with CPP, the peak LH concentration was mostly reached at 30 min (*n* = 18) and 45 min (*n* = 17) after gonadorelin injection and at 60 min (*n* = 30), 120 min (*n* = 23), and 180 min (*n* = 13) after triptorelin injection (Table 3). In more detail, the peak LH concentration was mostly reached at 30 min (*n* = 3), 60 min (*n* = 10), and 120 min (*n* = 22) in the triptorelin 120 min test. The peak LH concentration was mostly reached at 60 min (*n* = 20), 120 min (*n* = 1), and 180 min (*n* = 13) in the triptorelin 180 min test.

We divided girls with CPP into those in whom the peak LH level occurred <120 min or ≥120 min after triptorelin injection. There was no significant difference in the level of any clinical parameter or gonadotropin (Table 4).

**Figure 2:**
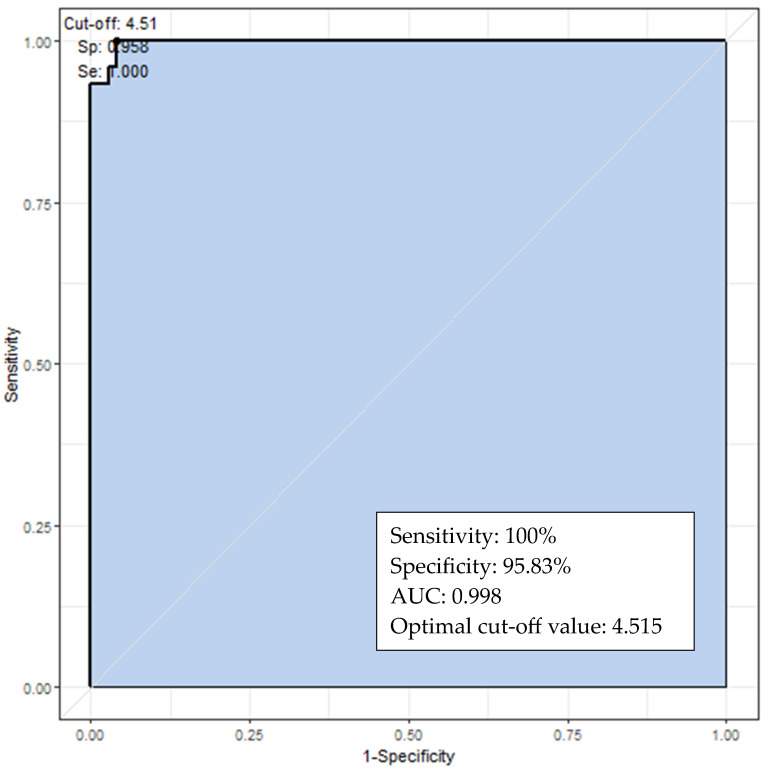
Receiver operating characteristic curves of the peak luteinizing hormone levels at 120 min used for diagnosing central precocious puberty via the triptorelin test.

We performed ROC curve analysis to evaluate the diagnostic utility of LH values for CPP in the overall triptorelin test group. A peak LH concentration of ≥4.52 IU/L at 120 min had the optimal diagnostic accuracy for CPP, with a sensitivity and specificity of 100% and 95.83%, respectively (Figure 2). The gonadorelin test group was subjected to ROC analysis; the optimal LH cutoff at 45 min was 4.88 IU/L, with a sensitivity and specificity of 100%.

## 4. Discussion

This study investigated the diagnostic utility of the triptorelin test in girls with CPP compared to the conventional gonadorelin test. To our knowledge, this is the first such study conducted in the Republic of Korea. The triptorelin test is as effective as the classic gonadorelin-based GnRH stimulation test in confirming the activation of the HPG axis, and it is an appropriate method to apply to the diagnosis of CPP and IPT in girls.

The peak LH concentration was reached later in the triptorelin test group than in the gonadorelin test group. In the gonadorelin test group, all girls with CPP showed a peak LH response at 30 to 60 min after intravenous gonadorelin injection. In the triptorelin test group, most girls with CPP showed a peak LH response at >120 min (n = 38). Gonadorelin and triptorelin can be used for diagnosing CPP. However, the time taken to reach the peak LH level differs between the two drugs due to variations in the route of administration (intravenous and subcutaneous, respectively) and pharmacokinetics.

Handelsman et al. [16] reported that when a single dose of GnRH was administered intravenously and subcutaneously, the plasma GnRH concentration increased earlier and was higher after intravenous injection than after subcutaneous injection (400 vs. 93.5 pg/mL, respectively); it also returned to the baseline value more quickly. After intravenous injection of GnRH, the plasma levels of GnRH peaked within 15 min and returned to the baseline levels at 60 min, whereas after subcutaneous injection of GnRH, the GnRH level peaked at 30–90 min and returned to the baseline level at 120 min. It was suggested that this reflected pharmacokinetic differences in the SC and IV routes of GnRH administration, attributable to prolonged and delayed GnRH absorption, as well as reduced GnRH bioavailability, on SC injection. In addition, drug levels following subcutaneous injections may be influenced by the injection volume, the tissue distribution of proteolytic enzymes, and the regional variation in blood flow.

Differences in the drug properties of gonadorelin and triptorelin may also affect the timing of LH peak concentrations. Lahlou et al. [17] reported that triptorelin has super-agonist effects compared to natural GnRH and gonadorelin because its receptor affinity is markedly higher than that of natural GnRH. In addition, triptorelin binding to gonadotroph membranes in vitro results in the release of 15 to 100 times more LH into the medium than that obtained with GnRH.

The clearance rate also plays a role; even intravenously administered triptorelin has a half-life several-fold higher than that of natural GnRH. Subcutaneous administration markedly slows the disappearance rate, as evidenced by a half-life that is 10-fold higher than that calculated from intravenous infusion. Thus, the action of subcutaneously administered triptorelin will be prolonged. In other words, triptorelin is a super-agonist that can sufficiently increase the plasma concentration of GnRH after subcutaneous injection and has a longer duration of action than gonadorelin. Thus, when using the triptorelin test, the LH level should be measured over a longer period of time in girls with suspected CPP.

In the United States, CPP is diagnosed when the peak LH level is >8 IU/L in a GnRH stimulation test and >5 IU/L in a GnRH agonist stimulation test using triptorelin. In the Republic of Korea, it is defined as a peak LH level of >5 IU/L in either the gonadorelin or triptorelin stimulation test [18,19]. Although the gonadorelin test is considered the gold standard for diagnosing CPP, the triptorelin test provides an alternative when gonadorelin is not readily available. The diagnostic value of triptorelin has been adequately substantiated in previous studies, but there is no consensus regarding the appropriate blood sampling intervals [11,12,13].

Zamboni et al. [20] were the first to use the triptorelin test in humans and highlighted its potential as a screening tool to distinguish gonadotropin deficiency from delayed puberty in boys. They administered 0.1 mg/m^2^ of triptorelin subcutaneously at 4 a.m.; blood samples for LH, FSH, and testosterone were collected 4 h after the injection. They based the timing of injection and blood sampling on a previous study involving five normal adult volunteers, which demonstrated that gonadotropin peaks 4 h after triptorelin administration.

Mason-Garcia et al. [21] administered 250 μg of [6-D-tryptophan]-LH-RH subcutaneously to five healthy adult men. Although the term is not widely used, [6-D-tryptophan]-LH-RH is a synonym of triptorelin. Following the administration of this analog, the baseline serum LH concentration increased from 11.8 ± 0.8 to 46.4 ± 7.9 mIU/mL after 90 min. LH levels remained elevated 24 h after administration (41.2 ± 7.2 mIU/mL).

Poomthavorn et al. [11] determined the optimal cutoff value for diagnosing CPP in girls using the triptorelin test. Blood samples were collected at 0, 30, 60, 90, and 120 min after a 100 µg subcutaneous triptorelin injection. Notably, peak LH levels were observed within 60 min. A peak LH level of 6 IU/L is considered an appropriate cutoff value for CPP diagnosis, with a sensitivity and specificity of 89.1% and 91.3%, respectively. In our study, blood samples were collected at 0, 30, 60, 90, and 120 min in the triptorelin 120 min group. Among all girls with CPP, 59.5% and 27% exhibited peak LH values at 120 and 60 min, respectively. We excluded girls aged > 9 years and those with pubic hair.

Freire et al. [12] compared the diagnostic accuracy of triptorelin to that of gonadorelin. In the case of triptorelin, blood samples were collected at 0, 3, and 24 h following a 100 µg subcutaneous injection. Notably, the peak LH level was observed at 3 h post-injection, and serum LH levels remained above baseline after 24 h. Based on a ROC curve analysis, a peak LH level of ≥7 IU/L is diagnostic for CPP, with a sensitivity and specificity of 76% and 100%, respectively. In our study, blood samples were collected at 0, 30, 60, 120, and 180 min in the triptorelin 180 min group. Among all girls with CPP, 54.1% and 31.5% exhibited peak LH values at 60 and 180 min, respectively, attributable to differences in the clinical progression rates. Most peak LH values occurred between 60 and 180 min.

Vukovic et al. [13] evaluated the diagnostic accuracy of the triptorelin test in girls with CPP and IPT using blood samples drawn at 0, 30, 60, 90, 120, and 180 min after the subcutaneous injection of 100 µg. The highest median LH level (25th–75th percentile) was reached at 180 min. In ROC analysis, a peak LH concentration ≥3.4 IU/L at 180 min provided the highest CPP diagnostic accuracy. We measured the peak LH values rather than the median values. As mentioned above, in the triptorelin 180 min test group, most girls with CPP exhibited peak LH values between 60 and 180 min. Our study measured the LH level with different sampling times in the two triptorelin tests (triptorelin 120 and 180 min tests). For the combined triptorelin test groups, the ROC curve revealed that a peak LH cutoff of ≥4.52 IU/L at 120 min was diagnostic of CPP, with a sensitivity and specificity of 100% and 95.83%, respectively. This suggests that a peak stimulated LH level ≥~4.5 IU/L after triptorelin injection may be indicative of CPP; alternatively, it may be necessary to extend the test time to 3 h after injection.

In this study, all girls with IPT exhibited breast development before the age of 8 years and BA advancement of > 1 year. Typically, IPT is considered a benign condition; breast development proceeds without significant HPG activation or BA advancement. Stanhope et al. [22] were the first to report girls with clinical findings intermediate between those of premature thelarche and CPP; the condition was termed the “thelarche variant” and was characterized by thelarche and growth rate acceleration. Su et al. [3] reported that some girls with IPT may also exhibit significantly advanced BA, which is strongly associated with obesity. Recently, rapidly progressing, slowly progressing, and non-progressive CPP have been distinguished. Based on such reports, it can be concluded that precocious puberty spans a spectrum ranging from isolated thelarche to rapidly progressive CPP; precocious puberty is not a single entity. Such individuals require long-term follow-up, but as our study was cross-sectional in nature, the girls were considered to have IPT. Further research is needed to ensure the accurate diagnosis and management of girls along the spectrum that runs from IPT to CPP.

This study had several limitations. The girls had undergone only one of the two tests, and normal prepubertal girls were not included in this study. Our study was retrospective and conducted after the replacement of gonadorelin with the triptorelin test due to a shortage of the former. Therefore, we could not perform both tests on the same subjects. Poomthavorn et al. [11] also lacked a normal control group and did not perform both tests on the same subjects. However, they proposed that the triptorelin test could be a useful alternative test. Likewise, in Freier et al. [12] and Vukovic et al. [13], no tests were performed on a normal control group. Conducting a study that involves both gonadorelin and triptorelin tests on the same patients could hold scientific value, but it necessitates careful consideration of its ethical feasibility. While the triptorelin test has shown high similarity to the gonadorelin test in previous research, making definitive conclusions without a complete pre-pubertal control group can be challenging. Ethical issues related to obtaining patient consent and the potential side effects of performing duplicate tests on the same patients need to be taken into account. The ethical aspects of the research should be carefully weighed, and in future studies, if possible, these ethical issues should be addressed to more thoroughly assess the reliability of the triptorelin test. In addition, the sample size of this study was relatively small, and all girls were Korean; thus, there are limitations in applying the results of this study to all ethnic groups. Despite these limitations, our study demonstrated that the triptorelin test can be used as a valid alternative to the gonadorelin test. In addition, our data support clinical application of the triptorelin test, shedding light on sampling times and the interpretation of the results, thus building on previous studies.

In conclusion, the triptorelin test is useful to confirm HPG activation for the diagnosis of CPP and is an alternative to the gonadorelin test. We suggest that a peak stimulated LH of ≥~4.5 IU/L 120 min after triptorelin injection should be considered indicative of CPP. Furthermore, for patients in whom CPP is strongly suspected, it may be valuable to extend the LH measurements to 3 h.

## 5. Conclusions

The triptorelin test reliably confirms hypothalamo-pituitary–gonadal activation in girls with suspected precocious puberty, and a diagnosis of CPP should be considered if the peak stimulated LH value at 120 min after triptorelin injection is ≥~4.5 IU/L.

## Figures and Tables

**Table 1:** Clinical characteristics of girls with central precocious puberty CPP and IPT.

	CPP (*n* = 111)	IPT (*n* = 109)	*p*-Value
Age (years)	8.11 ± 0.73	8.06 ± 0.63	0.608
Tanner stage	2.28 ± 0.45	2.19 ± 0.39	0.098
II	79	88	-
III	32	21	
BA–CA (years)	2.16 ± 0.74	1.96 ± 0.67	0.039 *
Height SDS	0.58 ± 0.92	0.75 ± 0.93	0.189
BMI SDS	0.51 ± 1.43	0.97 ± 1.30	0.014 *
Basal LH (IU/L)	0.63 ± 0.93	0.25 ± 0.13	<0.001 *
Basal FSH (IU/L)	2.93 ± 1.43	1.69 ± 0.72	<0.001 *
Peak LH (IU/L)	12.13 ± 11.44	3.12 ± 1.05	<0.001 *
Peak FSH (IU/L)	16.94 ± 5.76	13.84 ± 4.52	<0.001 *
Basal LH/FSH (IU/L)	0.22 ± 0.28	0.19 ± 0.18	0.4121
Peak LH/FSH (IU/L)	0.76 ± 0.63	0.23 ± 0.09	<0.001 *

Data are presented as mean ± standard deviation or the number of patients. Abbreviations: CPP, central precocious puberty; IPT, idiopathic premature thelarche; SDS, standard deviation score; BA–CA, bone age–chronological age ratio; BMI, body mass index; LH, luteinizing hormone. Statistical analysis was performed using the independent *t*-test. *, *p*-value < 0.05.

**Table 2:** Clinical characteristics of girls with CPP versus IPT according to the stimulant given.

	Triptorelin Test			Gonadorelin Test		
	CPP (*n* = 74)	IPT (*n* = 72)	*p*-Value	CPP (*n* = 37)	IPT (*n* = 37)	*p*-Value
Age (years)	8.11 ± 0.78	8.00 ± 0.56	0.477	8.11 ± 0.61	8.08 ± 0.73	0.880
Tanner stage	2.29 ± 0.46	2.00 ± 0.00	<0.001 *	2.27 ± 0.45	2.18 ± 0.39	0.414
II	52	58	-	27	31	-
III	22	14		10	6	
BA—CA (years)	2.09 ± 0.77	1.78 ± 0.56	0.033 *	2.30 ± 0.66	2.15 ± 0.72	0.352
Height SDS	0.74 ± 0.90	0.79 ± 0.97	0.721	0.27 ± 0.90	0.66 ± 0.87	0.064
BMI SDS	0.39 ± 1.59	0.85 ± 1.37	0.068	0.76 ± 1.01	1.22 ± 1.11	0.070
Basal LH (IU/L)	0.63 ± 1.09	0.20 ± 0.09	<0.005 *	0.62 ± 0.47	0.35 ± 0.16	<0.005 *
Basal FSH (IU/L)	2.93 ± 1.47	1.80 ± 0.73	<0.001 *	2.92 ± 1.36	1.46 ± 0.64	<0.001 *
Peak LH (IU/L)	11.94 ± 11.72	3.01 ± 1.02	<0.001 *	13.95 ± 13.44	3.33 ± 1.08	<0.001 *
Peak FSH (IU/L)	17.30 ± 5.82	14.06 ± 3.99	<0.001 *	16.22 ± 5.65	13.38 ± 5.53	0.042 *
Basal LH/FSH/ (IU/L)	0.19 ± 0.24	0.13 ± 0.08	0.036 *	0.27 ± 0.35	0.32 ± 0.25	0.521
Peak LH/FSH (IU/L)	0.73 ± 0.60	0.21 ± 0.08	<0.001 *	0.84 ± 0.69	0.25 ± 0.11	<0.001 *

Data are presented as means ± standard deviations or as numbers of patients. Abbreviations: CPP, central precocious puberty; IPT, idiopathic premature thelarche; SDS, standard deviation score; BA–CA, bone age–chronological age ratio; BMI, body mass index; LH, luteinizing hormone; * *p*-value < 0.05. Statistical analysis was performed using the independent *t*-test.

**Table 3:** Peak luteinizing hormone time according to the type of stimulant given to girls with central precocious puberty.

Blood Sampling Time	Gonadorelin	Triptorelin 120 min	Triptorelin 180 min
0 min	0 (0.0)		0 (0.0)
30 min	18 (48.7)	3(8.1)	3 (8.1)
45 min	17 (45.9)		-
60 min	2 (5.4)	10 (27.0)	18 (48.7)
90 min	0 (0.0)	2 (5.4)	-
120 min	-	22 (59.5)	3 (8.1)
180 min	-		13 (35.1)

Data are presented as *n* (%).

**Table 4:** Clinical characteristics of girls with CPP with peak LH levels around 120 min in the triptorelin test.

	<120 Min (*n* = 38)	≥120 Min (*n* = 36)	*p*-Value
Age (years)	8.22 ± 0.60	7.98 ± 0.93	0.188
Tanner stage	2.35 ± 0.48	2.22 ± 0.42	0.223
II	25	27	
III	14	8	
BA–CA (years)	1.97 ± 0.78	2.22 ± 0.75	0.174
Height SDS	0.64 ± 0.90	0.85 ± 0.89	0.307
BMI SDS	0.63 ± 0.91	0.16 ± 1.06	0.227
Basal LH (IU/L)	0.51 ± 0.82	0.75 ± 1.32	0.342
Basal FSH (IU/L)	2.74 ± 1.21	3.12 ± 1.69	0.267
Peak LH (IU/L)	13.34 ± 9.29	10.47 ± 13.81	0.295
Peak FSH (IU/L)	18.15 ± 5.88	16.40 ± 5.70	0.196
Basal LH/FSH (IU/L)	0.17 ± 0.15	0.21 ± 0.30	0.436
Peak LH/FSH (IU/L)	0.76 ± 0.45	0.69 ± 0.73	0.632

Data are presented as mean ± standard deviation or number of patients. Abbreviations: CPP, central precocious puberty; SDS, standard deviation score; BA–CA, bone age–chronological age ratio; BMI, body mass index; LH, luteinizing hormone. Statistical analysis was performed using the independent *t*-test.

## Data Availability

The data presented in this study are available on request from the corresponding author. The data are not publicly available due to to privacy of participants.
